# Spatiotemporal dynamics and serosurveillance landscape of brucellosis at the human-animal interface in the Chinese Southwest: A retrospective study

**DOI:** 10.1371/journal.pntd.0014358

**Published:** 2026-05-29

**Authors:** Qiuju Yang, Na Zhang, Yue Shi, Min Yuan, Canjun Zheng, Zhenjun Li, Zhiguo Liu

**Affiliations:** 1 Yunnan Provincial Key Laboratory for Natural Focal Disease Control and Prevention, Yunnan Institute of Endemic Diseases Control and Prevention, Kunming, the People’s Republic of China; 2 School of Public Health, Inner Mongolia Medical University, Hohhot, the People’s Republic of China; 3 National Key Laboratory of Intelligent Tracking and Forecasting for Infectious Diseases, National Institute for Communicable Disease Control and Prevention, Chinese Center for Disease Control and Prevention, Beijing, the People’s Republic of China; 4 Chinese Center for Disease Control and Prevention, Beijing, the People’s Republic of China; Colorado State University, UNITED STATES OF AMERICA

## Abstract

**Introduction:**

Human brucellosis is re-emerging in the Chinese Southwest, and its epidemic characteristics remain unclear.

**Methodology:**

Descriptive epidemiology, Joinpoint regression, spatial autocorrelation, serological surveillance and pathogen analysis were adopted to clarify the epidemiological evolution of the disease.

**Results:**

Between 2004 and 2024, 9,822 brucellosis cases occurred, dominated by 7,270 cases in Yunnan. The epidemic showed a persistent upward trend (AAPC ≥5.28, *P* < 0.05), and 51 cities/prefectures were identified with statistically significant increse (AAPC ≥ 9.30, *P* < 0.05). Serological surveillance of 36,459 individuals revealed a seropositivity rate of 2.13% (95%CI, 2.00%, 2.26%), identifying 1,831 new cases. Sheep/goats seropositivity at 1.02% (95%CI, 0.97%, 1.06%) and cattle at 0.45% (95%CI, 0.42%, 0.48%). Sustained elevated human incidence was validated by serological evidence, and seroprevalence trends in sheep/goats aligned closely with human epidemic dynamics. Human brucellosis has evolved from random to significantly geographically and spatial aggregated, spreading continuously with intensified clustering in central-eastern Yunnan, the Sichuan basin and Tibet’s Ngari-Qamdo axis, highlighting these as priority control zones. Human brucellosis has transitioned from sporadic to locally endemic, with phased progression and increasing geographical clustering recently. Human brucellosis in the Chinese Southwest is characterized by the persistent dominance of *B. melitensis* bv. 3, primarily represented by the co-circulating MLVA-11 genotypes GT116 and GT125, which form a stable, widely disseminated lineage across the region.

**Conclusion:**

From the re-emergence to persistent endemicity of human brucellosis in study area, the extensive distribution of *B. melitensis* with unique genotyping reveals a stable zoonotic cycle that requires coordinated regional control.

## Author summary

Brucellosis emerges as an increasing threat across the Chinese Southwest. From 2004 to 2024, 9,822 human brucellosis cases were reported. Transmission has shifted from sporadic cases to geographical clustering driven by the movement and illegal transfer of infected animals, with intensified epidemics in central and eastern Yunnan, the Sichuan Basin, and the Ngari–Qamdo corridor of Xizang. Serological testing revealed stratified seroprevalence across humans and livestock. The overall positive rate was 2.13% (95% CI: 2.00%–2.26%) in humans, 1.02% (95% CI: 0.97%–1.06%) in sheep and goats, and 0.45% (95% CI: 0.42%–0.48%) in cattle. Such serological evidence demonstrates sustained endemic circulation between livestock and local human populations. The ongoing resurgence of brucellosis in this region is predominantly attributed to *Brucella melitensis* biovar 3. Two dominant MLVA-11 genotypes (GT116 and GT125) exhibit extensive spatial distribution, reflecting a stable and long-standing local transmission cycle. The resurgence and persistent endemicity of brucellosis highlight the importance of One Health strategies in addressing neglected zoonotic threats.

## Introduction

Brucellosis remains a major global zoonotic threat, posing significant challenges to both public health systems and the economic sustainability of livestock industries [1]. Human infection primarily occurs through direct contact with infected animals or their products, consumption of contaminated dairy or meat, or inhalation of infectious aerosols [2]. Human brucellosis persists as a significant public health concern in endemic areas of Asia and Africa, its spread is facilitated by global mobility, and within mainland China, the epidemiological landscape has evolved, showing a rising trend and a spatial shift of the disease [3]. Reported cases of human brucellosis have traced a distinct path across mainland China: beginning in the northeast, expanding northwards, then turning southwest, and finally reaching Hainan Island in the far south [4].

Brucellosis has re-emerged in the Chinese Southwest, while clinical awareness among physicians and disease prevention knowledge among occupational populations remain insufficient [3]. Multiethnic communities in the Chinese Southwest, prevalent small-scale breeding, human-livestock cohabitation and raw livestock product consumption jointly fuel local disease endemicity [5]. From 2005 to 2021, Chinese human brucellosis incidence rose overall, and its accelerated growth in the South, Central, and Southwest regions shifted the epidemiological center of gravity from North to Southwest [5]. Although high-risk clusters of human brucellosis remain predominantly concentrated in the Chinese Northwest and Northeast, and the southern regions has experienced a marked rise, with its share of national cases increasing from 2.0% (4,310 cases) in 2019 to 5.38% (10,363 cases) in 2023 and notable new clusters emerging in Yunnan and Sichuan [6]. Serological surveillance at the human–animal interface serves as a core early indicator of disease occurrence and epidemics [7]. However, the spatiotemporal clustering, serological surveillance characteristics, and pathogen prevalence profiles of brucellosis in these regions remain poorly elucidated. In addition, brucellosis strains patients financially; a study of 563 patients in Chinese Xinjiang found most were middle/lower socioeconomic farmers/herders, with lower socioeconomic patients spending 37.23% of their income on care, highlighting financial pressure [8]. Accordingly, integrated analysis of its spatiotemporal trends, human–livestock interface serological data, and genetic diversity of dominant species is essential to develop targeted prevention and control strategies [9]. To address this research gap, the present study aims to characterize the epidemiological trends, spatial distribution patterns of human brucellosis, and the genetic diversity of *Brucella* strains in the Chinese Southwest from 2004 to 2024, using joinpoint regression, spatial autocorrelation analysis, and *Brucella* genotyping. The study seeks to generate targeted scientific evidence to optimize regional surveillance and control strategies, thereby mitigating the public health risks posed by this persistent zoonotic disease.

## Materials and methods

### Ethics statement

The study protocol received ethical review and approval from the Ethics Committee of the National Institute of Communicable Disease Control and Prevention. This study was based solely on retrospective analysis of anonymized surveillance data without direct participant involvement; therefore, individual informed consent was not required.

### Study area

Traditionally, based on geographical characteristics, climate differences, cultural and economic factors, and administrative management needs, Chinese 31 provinces/municipalities are divided into seven areas, among which the southwest region includes Sichuan Province, Chongqing Municipality, Guizhou Province, Yunnan Province, and Tibet Autonomous Region. Brucellosis is a reemerging infectious disease in the Chinese Southwest, with the first formally reported cases appearing in Tibet in 2004, followed by Chongqing, Sichuan, and Yunnan in 2006, and Guizhou in 2010. Administrative boundaries for mapping were sourced from a digital vector map (GS (2024) 0650) (S1 Fig).

### Study design

This study incorporated three core components: epidemiological analysis, serological surveillance, and pathogen molecular tracing in the Chinese Southwest. Epidemiological features were summarized using reported case counts and human brucellosis incidence. Joinpoint regression quantified overall and phased epidemic trends, while global and local spatial autocorrelation analyses assessed the spatial clustering of regional prevalence. Human and animal brucellosis seropositivity across multiple geographic scales was analyzed. Published pathogen detection and genotyping data were further synthesized to characterize the dominant *Brucella* species, geographic distribution, and genetic diversity of local epidemic strains

### Epidemic data collection

Epidemic data (2004–2024) on notified brucellosis cases and relevant incidence rates were sourced from the National Notifiable Disease Reporting System. This dataset consists of individual brucellosis cases, with relevant information submitted by attending doctors to the online National Notifiable Infectious Disease Reporting Information System of the Chinese Center for Disease Control and Prevention within 24 hours of diagnosis. All study data were anonymized to protect patient identities, data were managed in Excel 2021 (Microsoft, USA), and statistical analysis were performed in R software (version 4.4.1; Bell Laboratories, New Jersey, America).

### Serological surveillance data collection

Serological surveillance for humans and animals began in northern brucellosis-endemic areas in 2004 and expanded to the Chinese Southwest in 2006; only southwestern regional data were included in this study. Continuous human serological surveillance has been conducted locally since 2006, with one site per region from 2006–2017 and two fixed sites across five regions during 2018–2024 (Table A in S1 Table). Animal surveillance showed phased coverage changes. Sites were limited to Sichuan (1–3 sites) in 2006–2010 and 2018, then concentrated in Sichuan and Chongqing (2–3 sites) from 2020 to 2022. During 2023–2024, surveillance expanded to Sichuan, Chongqing, Yunnan and Guizhou, with two permanent sites in Chongqing and 6–7 regional sites in total (Table B in S1 Table).

All serological surveillance activities were carried out in strict accordance with the diagnostic criteria for human brucellosis issued in mainland China [10]. Strict quality control covering standardized serological assays, specimen management and instrument calibration was performed to ensure reliable epidemiological and serological data. At the surveillance sites, human and livestock blood samples were randomly collected per the surveillance protocol and transported to the local CDC laboratory as required. The Rose Bengal Plate Test (RBPT) was used for preliminary brucellosis screening, and the Serum Agglutination Test (SAT) for confirmation, with a diagnostic standard of serum agglutination titer ≥1:100. The 95% confidence interval was calculated following previously reported method [11] to estimate the reliability of the measured data.

### Temporal trend analysis of human brucellosis in the Chinese Southwest from 2004 to 2024

Trend characterization for the 2004–2024 incidence employed a Joinpoint regression analysis (Joinpoint Regression Program, version 5.2.0.2) [12] was performed to detect epidemic trends, with the maximum number of joinpoints set per software guidelines [13]. Briefly, the grid search method (GSM) establishes all possible interval segment function joinpoints (i.e., joinpoints) and calculates the corresponding sum of squares errors (SSE) and mean squared errors (MSE) for each possible scenario. The grid point that yields the smallest MSE is selected as the joinpoint for the segment function. Monte Carlo permutation test is the default model selection method in the software. Both segment-specific annual percentage changes (APC) and the overall average annual percentage change (AAPC) were computed to precisely quantify trend dynamics.

### Spatial autocorrelation analysis of human brucellosis in the Chinese Southwest from 2004 to 2024

Spatial autocorrelation was assessed at the county level using the inverse distance (Euclidean) method follow the previously reported [14]. Global clustering was evaluated with Moran’s I, where a coefficient significantly >0 indicates positive autocorrelation (clustering of similar values), < 0 indicates negative autocorrelation (dispersion), and ≈0 suggests randomness; significance was determined via Z-test (α = 0.05). Local patterns were analyzed using Local Indicators of Spatial Association (LISA) [15], identifying statistically significant clusters categorized as high-high (areas of high incidence surrounded by high incidence); high-low (high incidence adjacent to low incidence); low-high (low incidence surrounded by high incidence); and low-low (areas of low incidence bordered by low incidence).

### The distribution, genetic diversity and population structure of *Brucella* from the Chinese Southwest

To systematically characterize the prevalence and genetic diversity of *Brucella* in a given region, *Brucella*, Multiple-Locus Variable-Number Tandem-Repeat Analysis (MLVA), Multilocus Sequence Typing (MLST), Whole Genome Sequencing (WGS), and the names of each of the five regions were selected as the primary research items and presented in the English-published SCI study. Key indicators were extracted and organized, including the species/biovar profile of *Brucella*, the count of strains, the major circulating MLVA-11 genotypes, the geographic distribution of strains in the study area, and the time span of the study. All extracted data were cross-validated by two independent researchers, and any discrepancies were resolved through consultation with the corresponding authors of the original literature. In addition, MLSTdata based on nine loci of 1,309 *B. melitensis* strains from mainland China and Southeast Asia (S2 Table) were retrieved from the PubMLST database, and a minimum spanning tree (MST) based on the sequence types (STs) was constructed using GrapeTree 2.0 software [16] based on the MSTreeV2 algorithm to visualize their genetic relationships.

## Results

### General epidemic profile of human brucellosis in the Chinese Southwest, 2004–2024

From 2004 to 2024, a total of 9,822 human brucellosis cases were reported in the Southwest. The case distribution by province is as follows: 7, 270 cases in Yunnan, 1,307 in Guizhou, 657 in Chongqing, 441 in Sichuan, and 147 in Tibet. The corresponding average annual incidence rates per 100,000 population were 0.53/100,000 in Yunnan, 0.22/100,000 in Guizhou, 0.10/100,000 in Chongqing, 0.11/100,000 in Sichuan, and 1.42/100,000 in Tibet. A total of 36,459 individuals were surveyed, with 47,657 serological tests conducted. Among these, 1,017 tested positive, yielding a seropositivity rate of 2.13% (1,017/47,657) (95%CI, 2.00%, 2.26%). A total of 1,831 new cases were identified. Regarding animal surveillance, 217,464 sheep/goats were tested, of which 2,210 were seropositive, resulting in a sheep/goats seropositivity rate of 1.02% (95%CI, 0.97%, 1.06%). Among 174,220 cattle tested, 784 were seropositive, giving a cattle seropositivity rate of 0.45% (95%CI, 0.42%, 0.48%).

### Regional incidence dynamic of human brucellosis in the Chinese Southwest, 2004–2024

**Fig 1:**
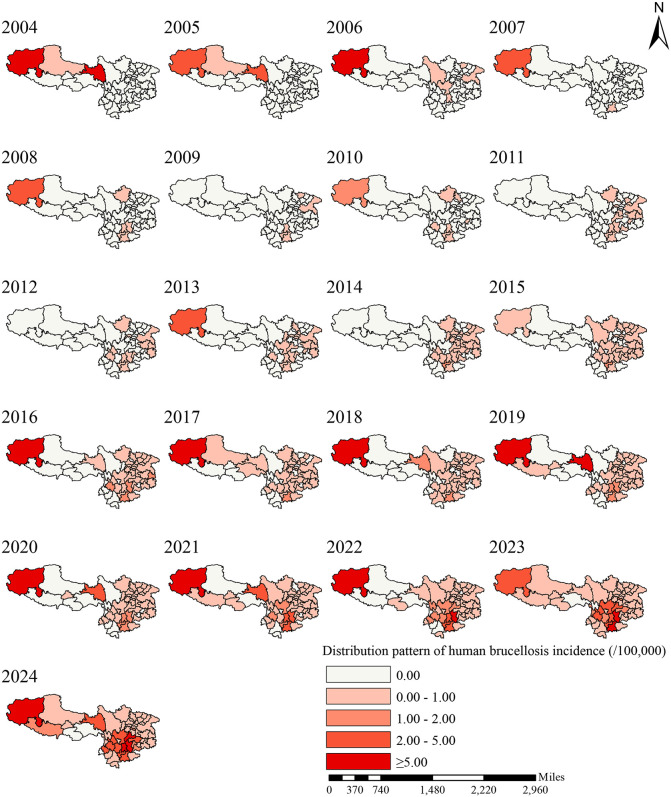
Distribution Pattern of Human Brucellosis Incidence in the Chinese Southwest, 2004-2024. Note: The figure illustrates the spatial distribution and temporal evolution of human brucellosis incidence in the Chinese Southwest (Tibet, Yunnan, Sichuan, Guizhou) from 2004 to 2024. Color intensity represents incidence levels, showing the transition from sporadic to widespread transmission over the study period. Data source: National Notifiable Disease Reporting System. Note: Map generation was completed in R. Chinese administrative boundary shapefiles originate from Tianditu National Geospatial Information Common Service Platform (https://cloudcenter.tianditu.gov.cn/administrativeDivision), official map review number GS (2024) 0650.

From 2004 to 2024, the epidemic in the Chinese Southwest evolved from sporadic distribution to widespread prevalence. The number of cities in the 0.00/100,000 interval (no case) sharply decreased from 49 to 7, a reduction of 85.7% (Fig 1); cities in the 0.00/100,000-1.00/100,000 interval increased from 1 to 18, reaching a peak of 18 in 2014 and then stabilizing; the 1.00/100,000-2.00/100,000 interval increased from 0 to 12, appearing for the first time in 2014 and continuing to rise; the 2.00/100,000-5.00/100,000 interval increased from 0 to 11, emerging in 2016 and rising rapidly thereafter; the ≥ 5.00/100,000 interval increased from 1 to 3, with Qamdo and Qujing entering this interval in 2024. The epidemic exhibited a clear stepwise escalation pattern, with many cities progressing from no cases to low, moderate, and even high incidence levels, indicating both the expanding scope and intensifying severity of the epidemic.

The spatial expansion of the epidemic exhibited a core-periphery diffusion pattern and regional clustering characteristics. In the early stage of the epidemic (2004–2013), the epidemic was primarily concentrated in Ngari Prefecture, Tibet. After 2014, cities with high incidence rates were observed in Yunnan, Sichuan, and Guizhou, leading to the formation of multiple epidemic zones. After 2020, central-eastern Yunnan (including Kunming, Qujing, Honghe, Chuxiong, *etc*.) formed a dense belt with an incidence rate in the 2.00/100,000–5.00/100,000 interval, while the Sichuan Basin developed a cluster with moderate incidence. Ngari Prefecture in Tibet remained a persistently high-incidence area, and Qamdo entered the high-incidence category in 2024. Ethnic autonomous prefectures (e.g., Chuxiong, Dali, Honghe) had earlier onset and greater incidence increase, showing obvious regional disparities. Ngari Prefecture maintained persistently high incidence; Qamdo and Qujing entered the ≥ 5.00/100,000interval in 2024, while Kunming, Honghe and Chuxiong approached the 5.00/100,000 threshold. Central-eastern Yunnan, the Sichuan Basin and the Ngari-Qamdo axis in Tibet form high-incidence zones.

### Joinpoint regression analysis of human brucellosis in the Chinese Southwest, 2004–2024

**Fig 2:**
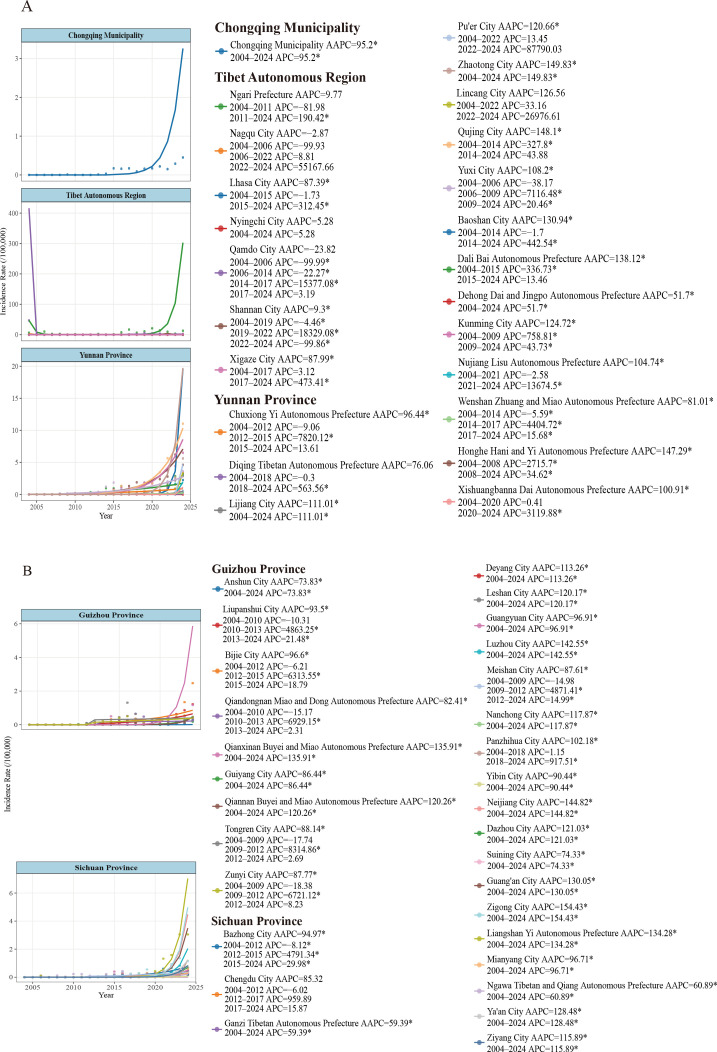
Joinpoint Regression Analysis of Human Brucellosis Incidence in the Chinese Southwest, 2004-2024. Note: Joinpoint regression analysis of human brucellosis incidence trends in the Chinese Southwest ((2A: Chongqing, Tibet, Yunnan) (2B: Guizhou, Sichuan,)) from 2004 to 2024. The analysis identifies significant turning points in incidence trends and calculates annual percentage changes (APC) for each segment. Notable regional variations are observed, with some areas showing significant increases (e.g., Dali (Yunnan Province) AAPC = 138.12*) while Nagqu City (Tibet) demonstrate declining trends (AAPC= -2.87). The plum symbolin the right plot indicate statistically significant joinpoints (*p* < 0.05) where trend directions changed substantially.

Joinpoint regression results indicated that nearly all prefecture-level regions in the Chinese Southwest recorded an AAPC of 5.28–154.44, with only Qamdo and Nagqu as exceptions. Statistically significant increasing trends were identified in 51 cities/prefectures (*P* < 0.05) (S3 Table). In addition, significant regional heterogeneity was observed, Sichuan Province had 20 cities/prefectures with significant upward trends, making it the most concentrated area of increase. Yunnan Province had 14 cities/prefectures with significant upward trends, AAPC values ranged from 51.70 to 148.10 (Fig. 2A). In Guizhou Province, nine regions showed significant increase, particularliy in Qiannan (AAPC=120.06), and Qianxinan (AAPC=135.91) (Fig. 2B). In the Tibet Autonomous Region, five cities/ prefectures demonstrated significant upward trends (AAPC = 5.28 -87.99); two regions declined,but the AAPC was not statistically significant.

APC analysis verified a substantial rising trend across 2004–2024 (Fig 2 (A+B) and S4 Table). Yunnan Province, a high-incidence hotspot, experienced sharp growth beginning in 2014 (APC = 76.04, *P* < 0.01) and drove the overall epidemic rebound. Sichuan’s incidence fluctuated at low levels before 2014, Chongqing entered an epidemiological plateau in 2019, while Guizhou Province recorded a steady, slow increasing trend. Divergent turning points among these regions highlighted distinct spatial heterogeneity.

Joinpoint regression further revealed obvious prefecture-level disparities across the Chinese Southwest. Qujing and Kunming in Yunnan presented continuous significant increases with APC values of 327.80 (2004–2014, *P* < 0.05) and 758.81 (2004–2009, *P* < 0.05), respectively, serving as core regions for the recent resurgence. Conversely, Bazhong City in Sichuan maintained mild undulation before 2012 (APC = −8.12, *P* > 0.05), and Kaizhou District of Chongqing sustained stable low endemicity from 20004 onward (APC = 95.20, *P* > 0.05). Two segmented significant trends were also found in Shannan, Tibet: in 2004-2019 (APC = -4.46, *P* > 0.05) and in 2022-2024 (APC = −99.86, *P* > 0.05).

### Global spatial autocorrelation analysis of brucellosis in the Chinese Southwest, 2004–2024

Global spatial autocorrelation revealed obvious staged shifts in spatial clustering patterns. During the early study period (2004–2008), the spatial distribution was largely random, with no significant clustering observed except for weak but statistically significant spatial autocorrelation in 2006 (Moran’s I = ‑0.006, *p* = 0.015) and 2007 (Moran’s I = ‑0.006, *p* = 0.011) (S5 Table). A clustering‑emergence phase occurred from 2009 to 2011, during which significant spatial aggregation appeared in 2009 (Moran’s I = 0.099, *p* = 0.004) and 2011 (Moran’s I = 0.014, *p* = 0.014). From 2012 to 2022, the level of spatial autocorrelation fluctuated considerably, but most years did not reach statistical significance. In the most recent two years (2023–2024), the spatial clustering trend intensified markedly, with 2023 showing the clustering pattern (Moran’s I = 0.134, *p* = 0.001) and 2024 remaining significant (Moran’s I = 0.082, *p* = 0.030).

### Local spatial autocorrelation analysis results for the Chinese southwest, 2004–2024

**Fig 3:**
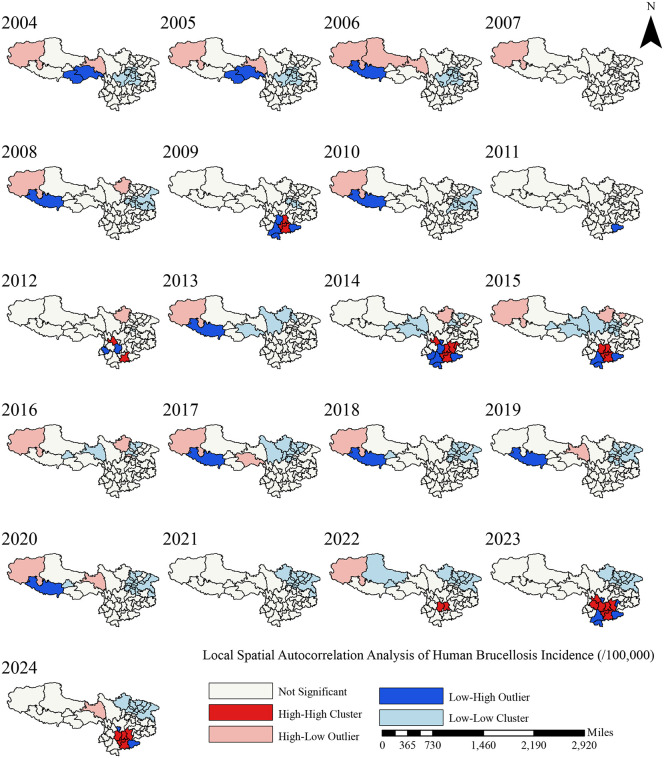
Local Spatial Autocorrelation Analysis of Human Brucellosis Incidence in the Chinese Southwest, 2004-2024. Note: Results of local spatial autocorrelation analysis (LISA) showing the spatial clustering patterns of human brucellosis incidence in the Chinese Southwest from 2004 to 2024. The map identifies: (1) High-High clusters (hot spots) indicating areas with significantly high incidence surrounded by similarly high areas; (2) Low-Low clusters (cold spots) showing areas with low incidence surrounded by low values; (3) spatial outliers including High-Low and Low-High areas. The analysis reveals significant geographical heterogeneity in disease distribution, with persistent hot spots in central Yunnan and western Sichuan, and cold spots in eastern Guizhou. All clusters shown are statistically significant (p < 0.05). Note: Map generation was completed in **R.** Chinese administrative boundary shapefiles originate from Tianditu National Geospatial Information Common Service Platform (https://cloudcenter.tianditu.gov.cn/administrativeDivision), official map review number GS (2024) 0650.

From 2004 to 2024, brucellosis in the Chinese Southwest exhibited marked spatiotemporal clustering. Initially sporadic in 2006, high-high (HH) clusters progressively expanded, rising from one in the early stage to seven in 2024 (Fig 3). Central, eastern and western Yunnan including Chuxiong, Kunming, Honghe, Qujing and Dali formed persistent clusters. By contrast, low‑low (LL) clusters followed a reverse trend, shrinking from an extensive distribution of 10–15 clusters to a limited range centered on the Sichuan Basin. The Sichuan Basin (Chengdu, Mianyang, Nanchong) sustained stable LL features, resulting in a striking high-low spatial contrast. Spatial outliers (HL, LH) were mainly concentrated in the border areas of Yunnan and Tibet, showing intermittent, marginal distribution.

### Serological dynamics of brucellosis in humans and livestock in Chinese Southwest, 2006–2024

Human brucellosis seropositivity exhibited a fluctuating upward trend. Seroprevalence stayed low from 2006 to 2010, ranging from 0.76% to 2.25%, before spiking to a peak of 9.17% in 2011. A secondary rise to 6.17% occurred in 2019, followed by sustained high levels at 0.99%–3.72% throughout 2020–2024 (Table 1). The overall cumulative seropositivity reached 2.13% (95% CI: 2.00%–2.26%). Significant temporal disparities in seroprevalence were detected across Chinese Southwest from 2006 to 2024 (*P* < 0.001).

**Table 1:** Serological dynamics of human brucellosis in the Chinese Southwest, 2006–2024.

Year	No. of individuals surveyed (persons)	No. of blood tests (persons)	No. of positives (persons)	No. of negative (persons)	Seropositivity rate (%)	LCL	UCL
2006	2,995	1,472	14	1,458	0.95	0.46%	1.45%
2007	2,995	1,157	13	1,144	1.12	0.52%	1.73%
2008	4,062	1,076	17	1,059	1.58	0.83%	2.33%
2009	2,360	488	11	477	2.25	0.94%	3.57%
2010	2,924	788	6	782	0.76	0.15%	1.37%
2011	3,946	938	86	852	9.17	7.32%	11.02%
2012	1,766	605	11	594	1.82	0.75%	2.88%
2013	1,597	552	14	538	2.54	1.22%	3.85%
2014	3,627	681	8	673	1.17	0.37%	1.98%
2015	1,576	512	7	505	1.37	0.36%	2.37%
2016	807	561	13	548	2.32	1.07%	3.56%
2017	839	537	17	520	3.17	1.68%	4.65%
2018	3,726	2,665	74	2,591	2.78	2.15%	3.40%
2019	3,239	3,081	190	2,891	6.17	5.32%	7.02%
2020	/	2,523	37	2,486	1.47	1.00%	1.94%
2021	/	22,224	221	22,003	0.99	0.86%	1.12%
2022	/	2,213	74	2,139	3.34	2.59%	4.09%
2023	/	2,657	95	2,562	3.58	2.87%	4.28%
2024	/	2,927	109	2,818	3.72	3.04%	4.41%
Total	36,459	47,657	1017	46,640	2.13	2.00%	2.26%

LCL:95% lower confidence limit, UCL = 95% upper confidence limit. “/”: No data.

Livestock surveillance data revealed divergent epidemiological trends among primary reservoir hosts. Sheep/goats maintained relatively high seropositivity in the early stage (0.78%–4.40%) (Table 2). Following the resumption of routine surveillance in 2018, their positive rates first declined sharply then rebounded steadily from 2021 to 2023, hitting 1.70% in 2023; the cumulative seroprevalence stood at 1.02% (95% CI: 0.97%–1.06%). By comparison, cattle seropositivity dropped substantially, falling from early high levels of 3.66%–7.71% to 0.02% in 2024, with a cumulative rate of merely 0.45% (95% CI: 0.42%–0.48%) (Table 3).

**Table 2:** Serological dynamics of Sheep/goat and Cattle brucellosis in the Chinese Southwest, 2006–2024.

Year	No. of Sheep /goat tested	No. of positive Sheep/goat	Sheep/goat seropositivity rate (%)	LCL	UCL	No. of Cattle tested	No. of Positive cattle	Cattle seropositivity rate (%)	LCL	UCL
2006	10,457	460	4.40%	4.01%	4.79%	3,118	114	3.66%	3.00%	4.31%
2007	7,710	332	4.31%	3.85%	4.76%	2,326	102	4.39%	3.55%	5.22%
2008	1,156	9	0.78%	0.27%	1.29%	913	42	4.60%	3.24%	5.96%
2009	695	9	1.29%	0.45%	2.14%	1,465	113	7.71%	6.35%	9.08%
2010	238	2	0.84%	0.32%	2.00%	1,897	79	4.16%	3.27%	5.06%
2018	6,139	0	0.00%	0.00%	0.00%	9,504	117	1.23%	1.01%	1.45%
2020	20,254	0	0.00%	0.00%	0.00%	5,523	28	0.51%	0.32%	0.69%
2021	25,728	28	0.11%	0.07%	0.15%	14,560	55	0.38%	0.28%	0.48%
2022	17,212	12	0.07%	0.03%	0.11%	1,992	4	0.20%	0.00%	0.40%
2023	68,982	1173	1.70%	1.60%	1.80%	25,273	107	0.42%	0.34%	0.50%
2024	58,893	185	0.31%	0.27%	0.36%	107,649	23	0.02%	0.01%	0.03%
Total	217,464	2210	1.02%	0.97%	1.06%	174,220	784	0.45%	0.42%	0.48%

No available data were recorded from 2011 to 2017 and 2019. LCL:95% lower confidence limit, UCL = 95% upper confidence limit.

**Table 3:** Species/biovar composition, MLVA genotype diversity, and geographic distribution of *Brucella melitensis* isolates from the Chinese Southwest based on Literatures.

Area	Region	No. of isolates	Species/biovar (number)	MLVA Genotypes*	Time span	Distribution of region	Ref.
The Chinese Southwest	Yunnan	514	*B. melitensis* bv.3 (n = 498)	MLVA-11: 15 genotypes (GT116 (n = 399), GT125 (n = 42)	2017-2023	12 prefectures/cities	[30]
			*B. melitensis* bv.1 (n = 16)	MLVA-16: 208 genotypes (96 shared, 112 unique)			
	Guizhou	83	*B. melitensis* bv. 3 (n = 80)and unknown(n = 1)	MLVA-11: 7 genotypes (GT116 (n = 44), GT125 (n = 27))	2009-2021	Eight cities	[31]
			*B. abortus* bv. 3 (n = 2)	MLVA-16: 49 genotypes (33 shared, 16 unique)			
	Sichuan	101	*B. melitensis* bv. 3 (n = 101)	MLVA-11: 8 genotypes (GT116 (n = 70), GT125 (n = 24))	2014-2021	16 prefectures/cities	[32]
				MLVA-16: 74 (17 shared, 54 unique)			

*: the numerical values represent the following: for MLVA-11, the total number of genotypes identified and the strain counts for predominant genotypes (GT116, GT125); for MLVA-16, the total number of genotypes and their distribution between shared (found in multiple samples/locations) and unique (found in single samples/locations) genotypes. Ref. as data source.

### County-level seroepidemiologic of human brucellosis in the Chinese Southwest, 2004–2024

Based on county-level surveillance data across the Chinese Southwest regions and surrounding provinces from 2006 to 2024, the human seroprevalence of brucellosis exhibited marked spatial heterogeneity at the county scale. The overall seropositivity ranged from 0.01% to 21.61%. High seroprevalence clustered in specific counties of Yunnan and Sichuan, whereas low-endemic areas were widely distributed across most monitored sites in Sichuan, Chongqing, Guizhou and Xizang. Seropositivity reached 21.61% (95% CI: 16.73%–26.49%) in Pixian County (Sichuan, 2011). In Yunnan (2019), rates were 19.29% (95% CI: 14.44%–24.14%) in Luliang County and 19.09% (95% CI: 15.66%–22.52%) in Shilin Yi Autonomous County. The rate fell to 10.53% (95% CI: 6.37%–14.69%) in Luliang County in 2023.

### County-level seroepidemiologic of livestock brucellosis in Chinese Southwest, 2004–2024

Based on county-level livestock surveillance data across the Chinese Southwest regions from 2006 to 2024, the seroprevalence of brucellosis in sheep/goats and cattle exhibited marked spatial and interspecific heterogeneity at the county scale. Overall seropositivity ranged from 0.01% to 5.79% for sheep/goats and from 0.00% to 7.17% for cattle. High seroprevalence in sheep/goats clustered in specific counties of Yunnan, whereas elevated cattle infection was concentrated in parts of Sichuan. Low endemicity in livestock brucellosis was widely observed across monitored sites in Chongqing. Sheep/goat seropositivity reached 5.79% (95% CI: 5.11%–6.47%) in Shilin Yi Autonomous County (Yunnan, 2023), followed by 5.59% (95% CI: 4.60%–6.58%) in Luliang County (Yunnan, 2023). Cattle seroprevalence peaked at 7.17% (95% CI: 5.81%–8.53%) in Ruoergai County (Sichuan, 2009), marking the highest infection level detected in cattle.

### Genetic diversity and population profile of *B. melitensis* circulating in the Chinese Southwest

**Fig 4:**
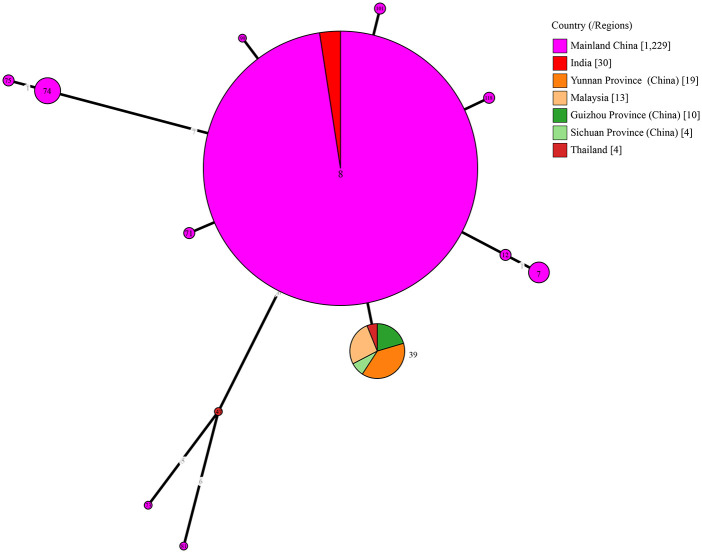
Transboundary distributions of ST39 *B. melitensis* in the Chinses Southwest and Southeast Asia. Note: The chord diagram displays the geographical distribution of reported ST39 *B. melitensis* isolates. The circular sectors represent different sequence types (STs), with the large central sector denoting ST8 isolates from mainland China (n = 1,229). The connecting chords between sectors indicate allelic differences among ST types, with line thickness proportional to genetic distance. The inset pie chart details 39 ST39 isolates from the Chinese Southwest and Southeast Asia regions, showing distribution by subregion: Yunnan, China (n = 19); Malaysia (n = 13); Guizhou, China (n = 10); Sichuan, China (n = 4); Thailand (n = 4).

The Chinese Southwest brucellosis exhibits distinctive epidemiological profiles, with *B. melitensis* biovar 3 as the persistent dominant pathogen (Table 3). MLVA‑11 genotyping revealed two prevalent genotypes (GT116 and GT125) belonging to a conserved genetic lineage, which reflects the long-term and large-scale spread of circulating strains. The MLVA-16 analysis demonstrated local genetic sharing within the Eastern Mediterranean lineage. MLST typing of 1,309 isolates detected 13 sequence types, of which ST8 was regionally predominant, followed by ST39, and the other 11 STs presented sporadically. Notably, *B. melitensis* ST39 is highly endemic to Chinese Southwest and neighboring Southeast Asia (Fig 4).

## Discussion

The present study integrates multi-source evidence to delineate the evolutionary of brucellosis in the Chinese Southwest across its resurgence, sporadic cases and localized endemicity, while clarifying epidemic dynamics to inform targeted regional control strategies [7]. The overall epidemic sustained expanding and worsening trends, showing a distinct stepwise escalation as multiple prefectures shifted from zero cases to high endemicity. This finding is consistent with the epidemiological characteristics observed in others southern provinces [17]. In Jiangsu Province, brucellosis incidence exhibited a sustained upward trend with distinct seasonal variation, peaking annually from April to June. From 2006 to 2021, the disease gradually spread from the northern and southern regions toward the central areas [18]. Rapid expansion of livestock husbandry and frequent cross-regional livestock transactions are key drivers of worsening brucellosis in the Chinese Southwest, while inadequate public awareness of prevention and control further exacerbates this endemic threat [19].

Correspondingly, the Chinese Southwest showed 28 prefectures exhibiting statistically significant positive AAPC; this trend aligns with the national epidemiological trend, counties (districts) with incidence rates exceeding 10/100,000 expanded geographically from northwestern pastoral regions to southern areas and from rural to urban settings, while emerging clusters have been identified in Yunnan, Guangdong, and Xizang [20]. Joinpoint regression analysis of human brucellosis in mainland China (2019–2023) revealed an upward trend in incidence rate across 29 provincial-level administrative divisions (PLADs), with particularly rapid increases observed in most southern regions, including the Southwest provinces (Yunnan, Guizhou, Sichuan) [6]. Overall, brucellosis in the Chinese Southwest has shifted from relative randomness to marked geographical aggregation, with this clustering trend intensifying in recent years.

The sustained rising incidence of human brucellosis is closely correlated with the expansion of livestock farming across the Chinses Southwest. Since 2000, sheep/goats farming in the Southwest region (Sichuan, Yunnan, Guizhou, Tibet) has generally shown a growth trend. Although the proportion of large-scale farming has increased from less than 20% to over 40%, individual farming still dominates, and regional development remains uneven [21]. The formation of core farming belts in the Sichuan Basin and central Yunnan has posed significant challenges for epidemic prevention and control. Previous analyses indicate that, since 2000, there has been a persistent increase in the frequency of cluster cases and number of reported livestock cases, with approximately 6.34% (34,070 of 537,797) of cases reported in the Chinese South from 2006 to 2021 [22]. Infected animals are a primary source of transmission, and their frequent movement, often involving illegal transfers, has contributed to the geographical expansion of the disease [23].

The animal surveillance data provide a plausible explanation for the sustained human brucellosis incidence in the Chinese Southwest. A meta-analysis indicates a concerning prevalence of yak brucellosis, with an overall pooled prevalence of 8.39%, reaching as high as 11.1% in the Chinese Southwest [24]. A systematic review and meta-analysis covering 2000–2018 demonstrated that the national brucellosis prevalence in sheep and goat flocks in mainland China exhibits distinct geographical heterogeneity, with the Chinese Southwest (Yunnan 5.30%, Guizhou 5.80%) showing a moderate but concerning seroprevalence [25]. These findings highlight the need for sustained vigilance to prevent imported brucellosis cases from evolving into local endemic transmission.

Serological surveillance is essential for detecting brucellosis occurrence and enabling early epidemic trend monitoring, as evidenced by the concurrent human and animal seropositivity recorded in the Chinese Southwest from 2006 to 2010. However, such surveillance approaches have prominent practical limitations, including fixed single‑site monitoring and insufficient representativeness of sampling and regional coverage. These drawbacks contribute to discrepancies between serological results and officially reported incidence. Such inconsistencies are further reflected in the divergent temporal trends of seropositivity between human populations and small ruminants. This deficiency pattern consistent with Asia and Africa where human brucellosis rebound contrasts with limited animal surveillance data [26]. These findings collectively demonstrate that while serological surveillance is effective for initial detection, it becomes insufficient once endemicity is established, particularly due to surveillance gaps and data inconsistencies. We recommend establishing an integrated surveillance system incorporating molecular testing in high-risk regions, alongside expanded surveillance coverage and larger sample sizes to improve the accuracy of epidemic risk assessment. These targeted measures will help mitigate prevalent surveillance gaps.

Human brucellosis in the Chinese Southwest is characterized by the sustained dominance of *B. melitensis* biovar 3 during 2009–2023, MLVA-16 analysis revealed both domestic and international genotype sharing Eastern Mediterranean lineage. This regional observation is consistent with the documented national expansion of *B. melitensis*. Molecular evidence indicates that southern strains share common geographic origins with northern strains and likely descended from northern ancestors [27]. These findings highlight the need for strengthened molecular surveillance to monitor the dissemination of *Brucella* strains. Importantly, the widespread distribution of *B. melitensis* ST39 across the Chinese Southwest and Southeast Asian regions is driven by contiguous border terrain, analogous climatic features and homogenized livestock breeding systems, collectively elevating the risk of cross-border zoonotic transmission [28]. The brucellosis epidemiological patterns identified in the Chinese Southwest provide evidence for targeted prevention across Southeast Asia.

Brucellosis poses substantial global challenges to surveillance, prevention and control as a core priority under the One Health framework, whose effective containment relies on cross-sectoral coordination across human, animal and environmental dimensions through integrated human-animal serological monitoring, cross-departmental data sharing, improved stakeholder communication, enhanced laboratory capacity, standardized veterinary management, targeted vaccination, environmental surveillance and strengthened professional prevention capabilities [29]. We recommend promoting the establishment of a joint regional surveillance and data sharing system in the Chinese Southwest, and taking timely response and control measures to block the chain of disease transmission and prevent imported infection sources from evolving into regional endemicity.

This study provides valuable insights into brucellosis epidemics, and several limitations should also be acknowledged. Epidemic trend analysis relied on passive surveillance data, while regional disparities in surveillance intensity, diagnostic capabilities and reporting completeness across cities/prefectures may compromise data accuracy. Furthermore, severe deficiencies in animal surveillance data restrict accurate interpretation of local epidemiological contexts. Limited molecular evidence from published literature only offers preliminary clues, and additional sampling and molecular evidence are warranted. Strengthened serological testing, field pathogen sampling and whole-genome surveillance across the human-animal interface are essential to clarify the transmission patterns of brucellosis.

## Conclusion

This study compiled a comprehensive epidemiological atlas of brucellosis in the Chinese Southwest. From 2004 to 2024, human brucellosis in this region has transitioned from sporadic cases to persistent endemicity, accompanied by substantial geographical expansion and widespread transmission. The epidemiological and transmission characteristics identified herein provide robust evidence to strengthen brucellosis surveillance and targeted interventions across the Chinese Southwest, and facilitate the formulation of cross-border coordinated prevention and control strategies. Future research should integrate multidimensional data from epidemiological investigations, bacteriological detection and molecular genetic analysis to systematically clarify regional epidemic profiles and cross-border epidemiological linkages. Such targeted measures are indispensable for blocking pathogen transmission cascades, curbing indigenous endemic persistence originating from cross-border introductions, and constructing a One Health-centred system for coordinated regional brucellosis mitigation.

## Supporting information

S1 Fig Geographical location of the study area in the Chinese Southwest region. The study area covers Sichuan Province, Chongqing Municipality, Guizhou Province, Yunnan Province, and Xizang Autonomous Region. Note: This map was created using R software. Asian continental and international boundary basemap data were retrieved from Natural Earth (medium scale). Chinese administrative boundary shapefiles were downloaded from the National Geospatial Information Common Service Platform (Tianditu, available at: https://cloudcenter.tianditu.gov.cn/administrativeDivision), with the official map review approval number GS (2024)0650.
(PNG)

S1 Table Distribution characteristics of serological surveillance sites for human and sheep/goat (S1-1) and cattle populations (S1-2). (XLSX)

S2 Table Population analysis of 1,309 *Brucella melitensis* strains from mainland China and Southeast Asia based on multilocus sequence typing (MLST). (XLSX)

S3 Table The average annual percent change (AAPC) results from joinpoint regression analysis of human brucellosis incidence trends in the Chinese Southwest during the period 2004–2024. (XLSX)

S4 Table Annual percent change (APC) values derived from joinpoint regression analyses of human brucellosis incidence trends in the Chinese Southwest, 2004–2024. (XLSX)

S5 Table Global spatial autocorrelation analysis results for the Chinese Southwest from 2004 to 2024. (XLSX)
