# Supplementary figures and images for: Spatiotemporal dynamics and serosurveillance landscape of brucellosis at the human-animal interface in the Chinese Southwest: A retrospective study
Source: PLoS Negl Trop Dis. 2026 May 29;20(5):e0014358. doi: 10.1371/journal.pntd.0014358 (PMC13241005; doi:10.1371/journal.pntd.0014358)

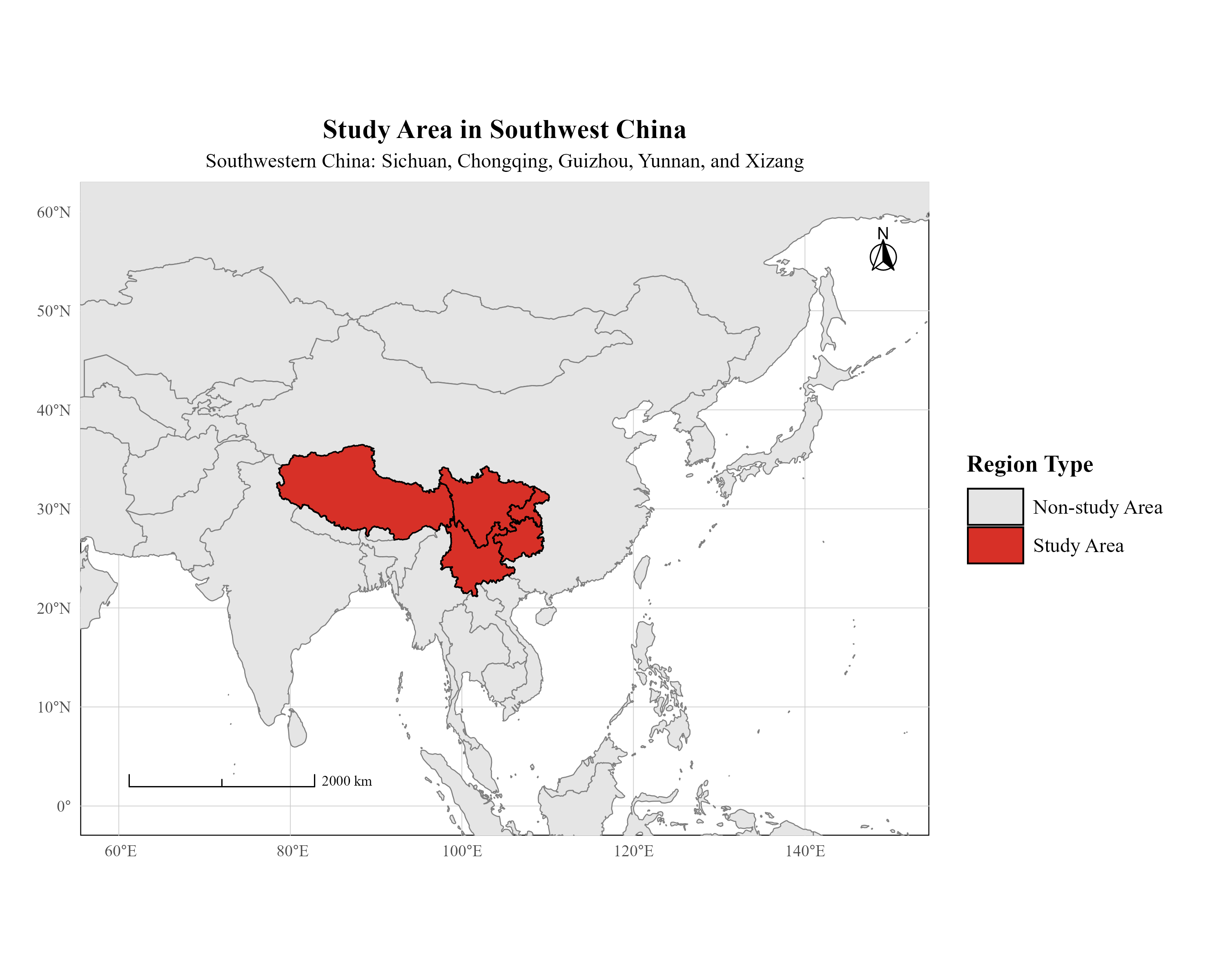

Supplement: S1 Fig — The study area covers Sichuan Province, Chongqing Municipality, Guizhou Province, Yunnan Province, and Xizang Autonomous Region. Note: This map was created using R software. Asian continental and international boundary basemap data were retrieved from Natural Earth (medium scale). Chinese administrative boundary shapefiles were downloaded from the National Geospatial Information Common Service Platform (Tianditu, available at: https://cloudcenter.tianditu.gov.cn/administrativeDivision), with the official map review approval number GS (2024)0650. (PNG) [file pntd.0014358.s001.png]
